# *rac*-2-Hydroxy-2,8-dimethyl-4-morpho­linoethyl-1-thia-4-aza­spiro­[4.5]decan-3-one

**DOI:** 10.1107/S1600536808022447

**Published:** 2008-07-23

**Authors:** Mehmet Akkurt, Şerife Pınar Yalçın, Nalan Terzioğlu Klip, Orhan Büyükgüngör

**Affiliations:** a Department of Physics, Faculty of Arts and Sciences, Erciyes University, 38039 Kayseri, Turkey; b Department of Pharmaceutical Chemistry, Faculty of Pharmacy, İIstanbul University, Beyazıt 34116, Istanbul, Turkey; c Department of Physics, Faculty of Arts and Sciences, Ondokuz Mayıs University, 55139 Samsun, Turkey

## Abstract

The title compound, C_16_H_28_N_2_O_3_S, is dimerized by inversion symmetry-related inter­molecular O—H⋯N hydrogen bonding, forming an *R*_2_^2^(16) motif. The dimers are also linked through inter­molecular C—H⋯O hydrogen bonding. The compound is chiral with a stereogenic centre located in the thia­zole ring, but in the crystal structure it forms a racemate. The thia­zole ring has an envelope conformation, while the cyclo­hexane and morpholine rings adopt chair conformations.

## Related literature

For general background, see: Andres *et al.* (2000 ▶); Çapan *et al.* (1999 ▶); Srivastava *et al.* (2005 ▶). For related literature and bond-length data, see: Akkurt *et al.* (2007 ▶); Akkurt, Yalçın, Güzeldemirci *et al.* (2008 ▶); Akkurt, Yalçın, Klip *et al.* (2008 ▶). For ring conformation puckering parameters, see: Cremer & Pople (1975 ▶). For hydrogen-bond motifs, see: Bernstein *et al.* (1995 ▶).
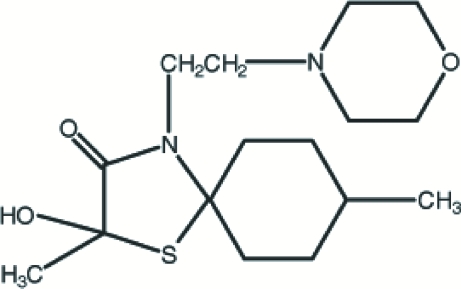


## Experimental

### 

#### Crystal data

C_16_H_28_N_2_O_3_S
*M* *_r_* = 328.47
Triclinic, 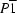

*a* = 8.0753 (4) Å
*b* = 10.2002 (5) Å
*c* = 11.8734 (6) Å
α = 82.467 (4)°
β = 71.487 (4)°
γ = 68.965 (4)°
*V* = 865.44 (8) Å^3^
*Z* = 2
Mo *K*α radiation
μ = 0.20 mm^−1^
*T* = 296 K
0.73 × 0.45 × 0.29 mm

#### Data collection

Stoe IPDS-2 diffractometer
Absorption correction: integration (*X-RED32*; Stoe & Cie, 2002 ▶) *T*_min_ = 0.867, *T*_max_ = 0.944
18220 measured reflections
3693 independent reflections
3319 reflections with *I* > 2σ(*I*)
*R*_int_ = 0.038

#### Refinement

*R*[*F* ^2^ > 2σ(*F* ^2^)] = 0.035
*wR*(*F* ^2^) = 0.093
*S* = 1.04
3693 reflections
199 parameters
H-atom parameters constrained
Δρ_max_ = 0.31 e Å^−3^
Δρ_min_ = −0.21 e Å^−3^

### 

Data collection: *X-AREA* (Stoe & Cie, 2002 ▶); cell refinement: *X-AREA*; data reduction: *X-RED32* (Stoe & Cie, 2002 ▶); program(s) used to solve structure: *SIR97* (Altomare *et al.*, 1999 ▶); program(s) used to refine structure: *SHELXL97* (Sheldrick, 2008 ▶); molecular graphics: *ORTEP-3 for Windows* (Farrugia, 1997 ▶); software used to prepare material for publication: *WinGX* (Farrugia, 1999 ▶) and *PLATON* (Spek, 2003 ▶).

## Supplementary Material

Crystal structure: contains datablocks global, I. DOI: 10.1107/S1600536808022447/kp2184sup1.cif

Structure factors: contains datablocks I. DOI: 10.1107/S1600536808022447/kp2184Isup2.hkl

Additional supplementary materials:  crystallographic information; 3D view; checkCIF report

## Figures and Tables

**Table 1:** Hydrogen-bond geometry (Å, °)

*D*—H⋯*A*	*D*—H	H⋯*A*	*D*⋯*A*	*D*—H⋯*A*
O3—H3⋯N2^i^	0.82	2.00	2.8104 (14)	169
C7—H7*B*⋯S1	0.97	2.82	3.2235 (18)	106
C14—H14*B*⋯O1^ii^	0.97	2.52	3.221 (2)	129

Symmetry codes: (i) 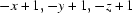; (ii) 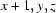.

**Fig. 1.**
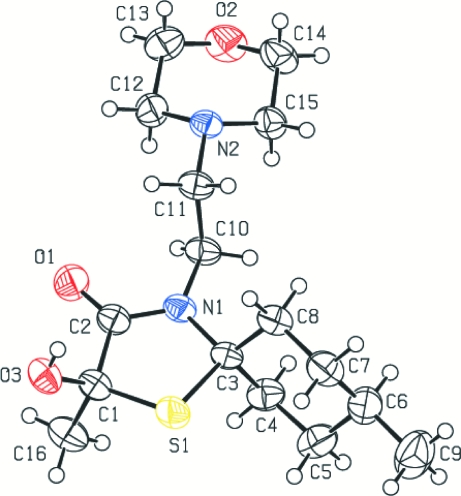
The ORTEP diagram of the title compound with the atom numbering scheme. Displacement ellipsoids for non-H atoms are drawn at the 50% probability level.

**Fig. 2.**
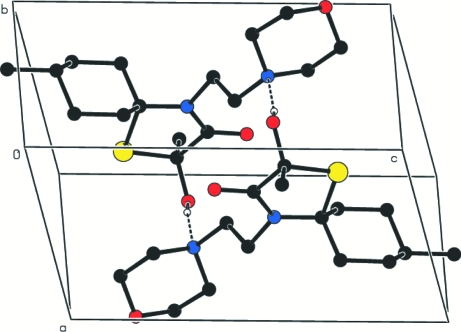
View of the dimer generated by inversion symmetry related O—H···N hydrogen bonds, forming a R22(16) motif. For clarity, H atoms not involved in hydrogen bonds have been omitted.

## supplementary crystallographic information

### Comment

Thiazolidinones and their spiroheterocyclic analogues have been reported to exhibit antibacterial (Andres *et al.*, 2000), antifungal (Çapan *et al.*, 1999) and antimycobacterial (Srivastava *et al.*, 2005) activity. In view of these observations, we synthesized the title spiro[4.5]decane derivative as a racemate and report its crystal structure.

In the title molecule (Fig. 1), the values of the geometric parameters are normal and comparable with those in the similar compound, 8-methyl-4-morpholinoethyl-1-thia- 4-azaspiro[4.5]decan-3-one (Akkurt, Yalçın, Klip *et al.*, 2008), which is a spiro[4.5]decane derivative.

The title compound is dimerized by inversion-symmetry related intermolecular O—H···N hydrogen bonds, forming an *R*_2_^2^(16) motif (Bernstein *et al.*,1995) (Fig. 2). The dimers are interlinked through intermolecular C—H···O hydrogen bonds. The compound is chiral with a stereogenic centre C1, in the thiazole ring. As the structure is centrosymmetric, racemate occurs in the crystal. The thiazole ring (C1–C3/S1/N1) has an envelope conformation on S1 [puckering parameters (Cremer & Pople, 1975): Q(2) = 0.1885 (12) Å, φ(2) = 5.7 (4) °]. The cyclohexane and morpholine rings (C3–C8) and (C12–C15/N2/O2) adopt chair conformations [puckering parameters: Q_T_ = 0.563 (2) Å, θ = 177.6 (2) °, φ = 58 (4) °, and Q_T_ = 0.576 (2) Å, θ = 0.28 (15) °, φ = 340 (60) °, respectively].

The structure is stabilized by intramolecular C—H···S and intermolecular O—H···N and C—H···O hydrogen bonds (Table 1, Fig. 2).

### Experimental

A mixture of morpholinoethylamin (5 mmol), 4-methyl cyclohexanone (5 mmol) and α-mercaptopropionic acid (20 mmol) in dry benzene (20 ml) was refluxed for 18 h using a Dean-Stark water separator. Excess solvent was evaporated *in vacuo*. The residue was taken up in chloroform. The chloroform layer was triturated with saturated NaHCO_3_ solution (2x) before drying over sodium sulfate and concentrated under reduced pressure to dryness. The crude product was triturated with diethyl ether several times and recrystallized from ethanol to yield racemic mixture as colourless prisms. IR (ν, cm^-1^): 1678 (C=O). ^1^H-NMR (δ, DMSO-d_6_, 400 MHz): 0.84 (3*H*, d, *J=*6.0 Hz, 8-CH_3_), 0.96–1.03 (1*H*, m, cycl. CH), 1.15–1.24 (1*H*, m, cycl.CH), 1.32–1.42 (3*H*, m, cycl.CH), 1.51–1.52 (1*H*, m, cycl.CH), 1.66 (2*H*, d, *J=*12.8 Hz, cycl.CH), 1.79 (1*H*, dd, *J=*12.8, 2.8 Hz, cycl.CH), 1.95- 2.10 (3*H*, m, SCHCH_3_), 2.33–2.44 (6*H*, m, morph.N—CH_2_), 3.30–3.37 (3*H*, m, SCH and N—CH_2_), 3.54 (4*H*, t, *J*=4.4 Hz, OCH_2_). LC—MS (m/*z*): 313 (*M*+1).

### Refinement

H atoms were positioned geometrically, with C—H = 0.98–0.96 Å, O—H = 0.82 Å and constrained to ride on their parent atoms. The thermal parameter of H-atoms of methyl and hydroxyl groups was taken 1.5 times of the parent atom, whereas for all other H-atoms it was taken 1.2 times of their parent atoms.

### Crystal data

**Table:**

C_16_H_28_N_2_O_3_S	*Z* = 2
*M**_r_* = 328.47	*F*_000_ = 356
Triclinic, *P*1	*D*_x_ = 1.260 Mg m^−^^3^
Hall symbol: -P 1	Mo *K*α radiation λ = 0.71073 Å
*a* = 8.0753 (4) Å	Cell parameters from 29023 reflections
*b* = 10.2002 (5) Å	θ = 2.1–27.4º
*c* = 11.8734 (6) Å	µ = 0.20 mm^−^^1^
α = 82.467 (4)º	*T* = 296 K
β = 71.487 (4)º	Block, colourless
γ = 68.965 (4)º	0.73 × 0.45 × 0.29 mm
*V* = 865.44 (8) Å^3^	

### Data collection

**Table:**

Stoe IPDS-2 diffractometer	3693 independent reflections
Monochromator: plane graphite	3319 reflections with *I* > 2σ(*I*)
Detector resolution: 6.67 pixels mm^-1^	*R*_int_ = 0.038
*T* = 296 K	θ_max_ = 26.9º
ω scans	θ_min_ = 2.1º
Absorption correction: integration(X-RED32; Stoe & Cie, 2002)	*h* = −10→10
*T*_min_ = 0.867, *T*_max_ = 0.944	*k* = −12→12
18220 measured reflections	*l* = −15→15

### Refinement

**Table:**

Refinement on *F*^2^	Secondary atom site location: difference Fourier map
Least-squares matrix: full	Hydrogen site location: inferred from neighbouring sites
*R*[*F*^2^ > 2σ(*F*^2^)] = 0.036	H-atom parameters constrained
*wR*(*F*^2^) = 0.093	*w* = 1/[σ^2^(*F*_o_^2^) + (0.0478*P*)^2^ + 0.1524*P*] where *P* = (*F*_o_^2^ + 2*F*_c_^2^)/3
*S* = 1.04	(Δ/σ)_max_ < 0.001
3693 reflections	Δρ_max_ = 0.31 e Å^−^^3^
199 parameters	Δρ_min_ = −0.21 e Å^−^^3^
Primary atom site location: structure-invariant direct methods	Extinction correction: none

### Special details

**Table:**

Geometry. Bond distances, angles *etc*. have been calculated using the rounded fractional coordinates. All su's are estimated from the variances of the (full) variance-covariance matrix. The cell e.s.d.'s are taken into account in the estimation of distances, angles and torsion angles
Refinement. Refinement on *F*^2^ for ALL reflections except those flagged by the user for potential systematic errors. Weighted *R*-factors *wR* and all goodnesses of fit *S* are based on *F*^2^, conventional *R*-factors *R* are based on *F*, with *F* set to zero for negative *F*^2^. The observed criterion of *F*^2^ > σ(*F*^2^) is used only for calculating -*R*-factor-obs *etc*. and is not relevant to the choice of reflections for refinement. *R*-factors based on *F*^2^ are statistically about twice as large as those based on *F*, and *R*-factors based on ALL data will be even larger.

### Fractional atomic coordinates and isotropic or equivalent isotropic displacement parameters (Å^2^)

**Table:**

	*x*	*y*	*z*	*U*_iso_*/*U*_eq_	
S1	0.33771 (4)	0.24227 (4)	0.79561 (3)	0.0486 (1)	
O1	0.45350 (15)	0.25388 (12)	0.45525 (9)	0.0555 (3)	
O2	1.23062 (15)	0.29531 (12)	0.15285 (9)	0.0597 (3)	
O3	0.11938 (12)	0.32395 (10)	0.65362 (9)	0.0480 (3)	
N1	0.59680 (14)	0.23670 (11)	0.59509 (9)	0.0389 (3)	
N2	0.90885 (14)	0.39307 (11)	0.34820 (9)	0.0372 (3)	
C1	0.28811 (17)	0.22315 (13)	0.65981 (12)	0.0417 (4)	
C2	0.45358 (17)	0.24085 (13)	0.55883 (11)	0.0408 (3)	
C3	0.58346 (16)	0.22120 (13)	0.72210 (10)	0.0369 (3)	
C4	0.7085 (2)	0.07643 (14)	0.75151 (12)	0.0482 (4)	
C5	0.7034 (2)	0.06187 (17)	0.88162 (14)	0.0583 (5)	
C6	0.7495 (2)	0.17691 (19)	0.92078 (13)	0.0587 (5)	
C7	0.6200 (2)	0.31982 (17)	0.89350 (13)	0.0541 (5)	
C8	0.62959 (19)	0.33651 (14)	0.76215 (12)	0.0445 (4)	
C9	0.7389 (3)	0.1587 (3)	1.05180 (17)	0.0881 (8)	
C10	0.76951 (17)	0.23709 (13)	0.50474 (11)	0.0413 (3)	
C11	0.75792 (17)	0.38482 (13)	0.45390 (11)	0.0408 (3)	
C12	0.90146 (19)	0.34106 (16)	0.24137 (11)	0.0471 (4)	
C13	1.0540 (2)	0.36400 (19)	0.13593 (12)	0.0584 (5)	
C14	1.2384 (2)	0.34737 (18)	0.25562 (13)	0.0554 (5)	
C15	1.09318 (17)	0.32359 (15)	0.36461 (11)	0.0432 (4)	
C16	0.2750 (2)	0.08031 (15)	0.65024 (15)	0.0561 (5)	
H3	0.12210	0.40260	0.65820	0.0720*	
H4A	0.66870	0.00470	0.73310	0.0580*	
H4B	0.83500	0.06140	0.70220	0.0580*	
H5A	0.58070	0.06390	0.93020	0.0700*	
H5B	0.79130	−0.02850	0.89480	0.0700*	
H6	0.87690	0.16960	0.87490	0.0700*	
H7A	0.65370	0.39320	0.91510	0.0650*	
H7B	0.49370	0.33050	0.94100	0.0650*	
H8A	0.54300	0.42730	0.74850	0.0530*	
H8B	0.75330	0.33370	0.71510	0.0530*	
H9A	0.82270	0.06800	1.06540	0.1320*	
H9B	0.61470	0.16630	1.09830	0.1320*	
H9C	0.77270	0.23040	1.07430	0.1320*	
H10A	0.79540	0.17570	0.44090	0.0500*	
H10B	0.87110	0.20060	0.53970	0.0500*	
H11A	0.64170	0.42750	0.43460	0.0490*	
H11B	0.75450	0.44050	0.51540	0.0490*	
H12A	0.78170	0.39070	0.22810	0.0570*	
H12B	0.91750	0.24180	0.25140	0.0570*	
H13A	1.05010	0.32880	0.06510	0.0700*	
H13B	1.03320	0.46390	0.12390	0.0700*	
H14A	1.21920	0.44710	0.24480	0.0670*	
H14B	1.36030	0.30050	0.26640	0.0670*	
H15A	1.11570	0.22360	0.37830	0.0520*	
H15B	1.09970	0.36120	0.43340	0.0520*	
H16A	0.17160	0.06840	0.71300	0.0840*	
H16B	0.38720	0.00790	0.65670	0.0840*	
H16C	0.25790	0.07430	0.57490	0.0840*	

### Atomic displacement parameters (Å^2^)

**Table:**

	*U*^11^	*U*^22^	*U*^33^	*U*^12^	*U*^13^	*U*^23^
S1	0.0372 (2)	0.0676 (2)	0.0380 (2)	−0.0232 (2)	−0.0003 (1)	−0.0024 (1)
O1	0.0540 (6)	0.0738 (7)	0.0406 (5)	−0.0258 (5)	−0.0106 (4)	−0.0033 (4)
O2	0.0495 (6)	0.0739 (7)	0.0429 (5)	−0.0201 (5)	0.0074 (4)	−0.0128 (5)
O3	0.0364 (5)	0.0458 (5)	0.0612 (6)	−0.0141 (4)	−0.0110 (4)	−0.0065 (4)
N1	0.0332 (5)	0.0452 (6)	0.0346 (5)	−0.0165 (4)	−0.0011 (4)	−0.0009 (4)
N2	0.0344 (5)	0.0407 (5)	0.0322 (5)	−0.0144 (4)	−0.0012 (4)	−0.0031 (4)
C1	0.0360 (6)	0.0429 (6)	0.0448 (7)	−0.0163 (5)	−0.0054 (5)	−0.0035 (5)
C2	0.0389 (6)	0.0406 (6)	0.0407 (6)	−0.0150 (5)	−0.0059 (5)	−0.0034 (5)
C3	0.0334 (5)	0.0390 (6)	0.0353 (6)	−0.0152 (5)	−0.0026 (4)	−0.0006 (4)
C4	0.0480 (7)	0.0408 (7)	0.0478 (7)	−0.0115 (6)	−0.0081 (6)	0.0007 (5)
C5	0.0593 (9)	0.0556 (8)	0.0498 (8)	−0.0133 (7)	−0.0140 (7)	0.0106 (6)
C6	0.0453 (7)	0.0845 (11)	0.0437 (7)	−0.0222 (7)	−0.0098 (6)	−0.0002 (7)
C7	0.0564 (8)	0.0637 (9)	0.0458 (7)	−0.0296 (7)	−0.0056 (6)	−0.0099 (6)
C8	0.0460 (7)	0.0430 (7)	0.0447 (7)	−0.0210 (5)	−0.0064 (5)	−0.0018 (5)
C9	0.0772 (13)	0.1301 (19)	0.0518 (10)	−0.0263 (12)	−0.0219 (9)	−0.0022 (10)
C10	0.0330 (6)	0.0421 (6)	0.0391 (6)	−0.0125 (5)	0.0022 (5)	−0.0012 (5)
C11	0.0355 (6)	0.0396 (6)	0.0387 (6)	−0.0126 (5)	0.0018 (5)	−0.0038 (5)
C12	0.0457 (7)	0.0579 (8)	0.0387 (6)	−0.0195 (6)	−0.0102 (5)	−0.0038 (5)
C13	0.0639 (9)	0.0734 (10)	0.0334 (7)	−0.0260 (8)	−0.0045 (6)	−0.0019 (6)
C14	0.0409 (7)	0.0703 (9)	0.0520 (8)	−0.0259 (7)	0.0014 (6)	−0.0080 (7)
C15	0.0355 (6)	0.0517 (7)	0.0395 (6)	−0.0150 (5)	−0.0048 (5)	−0.0055 (5)
C16	0.0533 (8)	0.0466 (8)	0.0683 (9)	−0.0239 (6)	−0.0077 (7)	−0.0058 (6)

### Geometric parameters (Å, °)

**Table:**

S1—C1	1.8295 (14)	C4—H4B	0.9700
S1—C3	1.8410 (14)	C5—H5A	0.9700
O1—C2	1.2197 (16)	C5—H5B	0.9700
O2—C13	1.412 (2)	C6—H6	0.9800
O2—C14	1.4204 (19)	C7—H7A	0.9700
O3—C1	1.3990 (17)	C7—H7B	0.9700
O3—H3	0.8200	C8—H8A	0.9700
N1—C3	1.4688 (15)	C8—H8B	0.9700
N1—C10	1.4659 (18)	C9—H9A	0.9600
N1—C2	1.3420 (19)	C9—H9B	0.9600
N2—C12	1.4633 (17)	C9—H9C	0.9600
N2—C15	1.4651 (19)	C10—H10A	0.9700
N2—C11	1.4636 (17)	C10—H10B	0.9700
C1—C2	1.535 (2)	C11—H11A	0.9700
C1—C16	1.520 (2)	C11—H11B	0.9700
C3—C8	1.524 (2)	C12—H12A	0.9700
C3—C4	1.5298 (19)	C12—H12B	0.9700
C4—C5	1.521 (2)	C13—H13A	0.9700
C5—C6	1.516 (2)	C13—H13B	0.9700
C6—C9	1.521 (2)	C14—H14A	0.9700
C6—C7	1.523 (2)	C14—H14B	0.9700
C7—C8	1.527 (2)	C15—H15A	0.9700
C10—C11	1.5297 (18)	C15—H15B	0.9700
C12—C13	1.513 (2)	C16—H16A	0.9600
C14—C15	1.506 (2)	C16—H16B	0.9600
C4—H4A	0.9700	C16—H16C	0.9600
			
S1···N1	2.6146 (11)	H5B···H9A	2.4800
S1···H5A	2.8900	H6···H8B	2.5600
S1···H7B	2.8200	H6···H16A^v^	2.5100
O1···O3	2.8913 (15)	H7A···H9C	2.5400
O1···C11	3.187 (2)	H7A···H14A^vi^	2.5400
O1···C14^i^	3.221 (2)	H7B···S1	2.8200
O1···C15^i^	3.230 (2)	H7B···O2^x^	2.8100
O2···N2	2.8364 (16)	H7B···H5A	2.5600
O3···C15^ii^	3.3942 (17)	H7B···H9B	2.5300
O3···O1	2.8913 (15)	H8A···H12A^ii^	2.5600
O3···N2^ii^	2.8104 (14)	H8B···O3^v^	2.7800
O1···H10A	2.5400	H8B···C10	2.7500
O1···H11A	2.6600	H8B···C11	3.0700
O1···H15B^i^	2.7500	H8B···H6	2.5600
O1···H16C	2.8000	H8B···H10B	2.3800
O1···H14B^i^	2.5200	H8B···H11B	2.4700
O1···H16B^iii^	2.8200	H8B···H14A^vi^	2.4400
O2···H9B^iv^	2.7900	H9A···H5B	2.4800
O2···H7B^iv^	2.8100	H9B···O2^x^	2.7900
O3···H15B^i^	2.6400	H9B···C14^x^	3.0700
O3···H8B^i^	2.7800	H9B···H5A	2.5000
O3···H14A^ii^	2.9100	H9B···H7B	2.5300
N1···S1	2.6146 (11)	H9B···H14B^x^	2.4800
N2···O2	2.8364 (16)	H9C···C12^xi^	3.0000
N2···O3^ii^	2.8104 (14)	H9C···H7A	2.5400
N2···H3^ii^	2.0000	H10A···O1	2.5400
C2···C11^ii^	3.5847 (18)	H10A···C12	2.8100
C8···C11	3.4925 (18)	H10A···H12B	2.2700
C11···C8	3.4925 (18)	H10B···C4	2.8100
C11···C2^ii^	3.5847 (18)	H10B···C8	2.9100
C11···O1	3.187 (2)	H10B···C15	2.7700
C14···O1^v^	3.221 (2)	H10B···H4B	2.2500
C15···O1^v^	3.230 (2)	H10B···H8B	2.3800
C15···O3^ii^	3.3942 (17)	H10B···H15A	2.3300
C2···H11A	2.8300	H11A···O1	2.6600
C4···H10B	2.8100	H11A···C2	2.8300
C8···H14A^vi^	2.8600	H11A···H12A	2.3700
C8···H10B	2.9100	H11A···C11^ii^	3.0600
C8···H11B	2.9700	H11A···H11A^ii^	2.4000
C10···H12B	2.8600	H11B···C8	2.9700
C10···H4B	2.8300	H11B···H8B	2.4700
C10···H15A	2.6800	H11B···H15B	2.5000
C10···H8B	2.7500	H12A···H11A	2.3700
C11···H8B	3.0700	H12A···H8A^ii^	2.5600
C11···H3^ii^	2.7100	H12B···C10	2.8600
C11···H11A^ii^	3.0600	H12B···H10A	2.2700
C12···H3^ii^	2.9300	H12B···H15A	2.4700
C12···H10A	2.8100	H13B···H14A	2.3400
C12···H9C^vii^	3.0000	H14A···H13B	2.3400
C14···H9B^iv^	3.0700	H14A···O3^ii^	2.9100
C14···H3^ii^	3.0800	H14A···C8^vi^	2.8600
C15···H10B	2.7700	H14A···H3^ii^	2.5900
C15···H3^ii^	2.7400	H14A···H7A^vi^	2.5400
H3···N2^ii^	2.0000	H14A···H8B^vi^	2.4400
H3···C11^ii^	2.7100	H14B···O1^v^	2.5200
H3···C12^ii^	2.9300	H14B···H9B^iv^	2.4800
H3···C14^ii^	3.0800	H15A···C10	2.6800
H3···C15^ii^	2.7400	H15A···H10B	2.3300
H3···H14A^ii^	2.5900	H15A···H12B	2.4700
H4A···H15A^viii^	2.5800	H15A···H4A^viii^	2.5800
H4B···C10	2.8300	H15B···O1^v^	2.7500
H4B···H10B	2.2500	H15B···O3^v^	2.6400
H5A···S1	2.8900	H15B···H11B	2.5000
H5A···H7B	2.5600	H16A···H6^i^	2.5100
H5A···H9B	2.5000	H16B···O1^iii^	2.8200
H5A···H5A^ix^	2.3300	H16C···O1	2.8000
			
C1—S1—C3	94.96 (6)	C6—C7—H7B	109.00
C13—O2—C14	109.74 (12)	C8—C7—H7A	109.00
C1—O3—H3	109.00	C8—C7—H7B	109.00
C2—N1—C10	118.36 (10)	H7A—C7—H7B	108.00
C3—N1—C10	121.36 (11)	C3—C8—H8A	109.00
C2—N1—C3	120.12 (11)	C3—C8—H8B	109.00
C11—N2—C15	113.24 (10)	C7—C8—H8A	109.00
C12—N2—C15	109.83 (11)	C7—C8—H8B	109.00
C11—N2—C12	113.29 (11)	H8A—C8—H8B	108.00
S1—C1—C2	104.53 (10)	C6—C9—H9A	109.00
S1—C1—C16	113.45 (10)	C6—C9—H9B	109.00
S1—C1—O3	110.65 (9)	C6—C9—H9C	109.00
O3—C1—C16	107.00 (12)	H9A—C9—H9B	110.00
C2—C1—C16	108.91 (11)	H9A—C9—H9C	109.00
O3—C1—C2	112.40 (11)	H9B—C9—H9C	109.00
O1—C2—C1	121.93 (14)	N1—C10—H10A	109.00
N1—C2—C1	113.93 (11)	N1—C10—H10B	109.00
O1—C2—N1	124.12 (13)	C11—C10—H10A	109.00
S1—C3—N1	103.79 (9)	C11—C10—H10B	109.00
S1—C3—C8	109.34 (9)	H10A—C10—H10B	108.00
N1—C3—C4	111.50 (10)	N2—C11—H11A	108.00
S1—C3—C4	110.49 (9)	N2—C11—H11B	108.00
C4—C3—C8	110.34 (12)	C10—C11—H11A	108.00
N1—C3—C8	111.20 (10)	C10—C11—H11B	108.00
C3—C4—C5	111.95 (11)	H11A—C11—H11B	107.00
C4—C5—C6	112.88 (13)	N2—C12—H12A	110.00
C5—C6—C7	109.51 (14)	N2—C12—H12B	110.00
C7—C6—C9	111.87 (16)	C13—C12—H12A	110.00
C5—C6—C9	110.62 (16)	C13—C12—H12B	110.00
C6—C7—C8	111.54 (12)	H12A—C12—H12B	108.00
C3—C8—C7	111.69 (12)	O2—C13—H13A	109.00
N1—C10—C11	111.71 (11)	O2—C13—H13B	109.00
N2—C11—C10	115.84 (11)	C12—C13—H13A	109.00
N2—C12—C13	109.15 (13)	C12—C13—H13B	109.00
O2—C13—C12	111.53 (12)	H13A—C13—H13B	108.00
O2—C14—C15	111.11 (14)	O2—C14—H14A	109.00
N2—C15—C14	109.61 (11)	O2—C14—H14B	109.00
C3—C4—H4A	109.00	C15—C14—H14A	109.00
C3—C4—H4B	109.00	C15—C14—H14B	109.00
C5—C4—H4A	109.00	H14A—C14—H14B	108.00
C5—C4—H4B	109.00	N2—C15—H15A	110.00
H4A—C4—H4B	108.00	N2—C15—H15B	110.00
C4—C5—H5A	109.00	C14—C15—H15A	110.00
C4—C5—H5B	109.00	C14—C15—H15B	110.00
C6—C5—H5A	109.00	H15A—C15—H15B	108.00
C6—C5—H5B	109.00	C1—C16—H16A	109.00
H5A—C5—H5B	108.00	C1—C16—H16B	109.00
C5—C6—H6	108.00	C1—C16—H16C	109.00
C7—C6—H6	108.00	H16A—C16—H16B	109.00
C9—C6—H6	108.00	H16A—C16—H16C	109.00
C6—C7—H7A	109.00	H16B—C16—H16C	109.00
			
C3—S1—C1—O3	−136.07 (10)	C12—N2—C15—C14	56.88 (15)
C3—S1—C1—C2	−14.85 (9)	C12—N2—C11—C10	71.48 (15)
C3—S1—C1—C16	103.66 (11)	S1—C1—C2—N1	12.26 (13)
C1—S1—C3—N1	13.84 (9)	O3—C1—C2—O1	−49.39 (17)
C1—S1—C3—C4	−105.81 (9)	O3—C1—C2—N1	132.32 (12)
C1—S1—C3—C8	132.57 (9)	C16—C1—C2—O1	69.00 (17)
C14—O2—C13—C12	−59.69 (17)	C16—C1—C2—N1	−109.30 (13)
C13—O2—C14—C15	59.60 (16)	S1—C1—C2—O1	−169.45 (11)
C2—N1—C10—C11	79.30 (14)	N1—C3—C8—C7	−178.85 (12)
C3—N1—C2—O1	179.78 (12)	C8—C3—C4—C5	52.97 (16)
C10—N1—C2—O1	−4.83 (19)	S1—C3—C8—C7	67.13 (14)
C3—N1—C10—C11	−105.38 (13)	N1—C3—C4—C5	177.06 (13)
C2—N1—C3—C8	−126.75 (13)	C4—C3—C8—C7	−54.59 (16)
C10—N1—C3—C8	58.01 (15)	S1—C3—C4—C5	−68.06 (15)
C10—N1—C2—C1	173.42 (10)	C3—C4—C5—C6	−54.55 (19)
C2—N1—C3—C4	109.65 (14)	C4—C5—C6—C7	55.23 (18)
C2—N1—C3—S1	−9.30 (13)	C4—C5—C6—C9	178.97 (16)
C10—N1—C3—S1	175.45 (9)	C5—C6—C7—C8	−56.25 (18)
C10—N1—C3—C4	−65.59 (15)	C9—C6—C7—C8	−179.25 (16)
C3—N1—C2—C1	−1.97 (16)	C6—C7—C8—C3	57.30 (18)
C11—N2—C12—C13	175.83 (12)	N1—C10—C11—N2	−169.01 (11)
C15—N2—C11—C10	−54.44 (15)	N2—C12—C13—O2	58.47 (17)
C15—N2—C12—C13	−56.45 (15)	O2—C14—C15—N2	−58.52 (16)
C11—N2—C15—C14	−175.38 (11)		

Symmetry codes: (i) *x*−1, *y*, *z*; (ii) −*x*+1, −*y*+1, −*z*+1; (iii) −*x*+1, −*y*, −*z*+1; (iv) *x*+1, *y*, *z*−1; (v) *x*+1, *y*, *z*; (vi) −*x*+2, −*y*+1, −*z*+1; (vii) *x*, *y*, *z*−1; (viii) −*x*+2, −*y*, −*z*+1; (ix) −*x*+1, −*y*, −*z*+2; (x) *x*−1, *y*, *z*+1; (xi) *x*, *y*, *z*+1.

### Hydrogen-bond geometry (Å, °)

**Table:**

*D*—H···*A*	*D*—H	H···*A*	*D*···*A*	*D*—H···*A*
O3—H3···N2^ii^	0.82	2.00	2.8104 (14)	169
C7—H7B···S1	0.97	2.82	3.2235 (18)	106
C14—H14B···O1^v^	0.97	2.52	3.221 (2)	129

Symmetry codes: (ii) −*x*+1, −*y*+1, −*z*+1; (v) *x*+1, *y*, *z*.
